# Risk-prediction nomogram for congenital heart disease in offspring of Chinese pregnant women

**DOI:** 10.1186/s12884-024-06708-4

**Published:** 2024-07-27

**Authors:** Pengfei Qu, Shutong Zhang, Jie Chen, Xiayang Li, Doudou Zhao, Danmeng Liu, Mingwang Shen, Hong Yan, Leilei Pei, Shaonong Dang

**Affiliations:** 1 Translational Medicine Center, Northwest Women’s and Children’s Hospital, No.1616 Yanxiang Road, Xi’an, Shaanxi 710061 China; 2 Department of Epidemiology and Health Statistics, School of Public Health, Xi’an Jiaotong University Health Science Center, No.76 Yanta West Road, Xi’an, Shaanxi 710061 China

**Keywords:** Congenital heart disease, Nomogram, Prediction model, Pregnant women, Chinese population

## Abstract

**Background:**

The identification and assessment of environmental risks are crucial for the primary prevention of congenital heart disease (CHD). We were aimed to establish a nomogram model for CHD in the offspring of pregnant women and validate it using a large CHD database in Northwest China.

**Methods:**

A survey was conducted among 29,204 women with infants born between 2010 and 2013 in Shaanxi province, Northwest China. Participants were randomly assigned to the training set and to the validation set at a ratio of 7:3. The importance of predictive variables was assessed using random forest. A multivariate logistic regression model was used to construct the nomogram for the prediction of CHD.

**Results:**

Multivariate analyses revealed that the gravidity, preterm birth history, family history of birth defects, infection, taking medicine, tobacco exposure, pesticide exposure and singleton/twin pregnancy were significant predictive risk factors for CHD in the offspring of pregnant women. The area under the receiver operating characteristic curve for the prediction model was 0.716 (95% CI: 0.671, 0.760) in the training set and 0.714 (95% CI: 0.630, 0.798) in the validation set, indicating moderate discrimination. The prediction model exhibited good calibration (Hosmer-Lemeshow χ^2^ = 1.529, *P* = 0.910).

**Conclusions:**

We developed and validated a predictive nomogram for CHD in offspring of Chinese pregnant women, facilitating the early prenatal assessment of the risk of CHD and aiding in health education.

## Introduction

Congenital heart disease (CHD) is a malformation caused by abnormal cardiovascular development in the fetus, potentially leading to miscarriage, stillbirth, and infant mortality, significantly impacting quality of life and posing a substantial disease burden [1]. CHD is the most prevalent type of congenital malformation, comprising approximately 30% of all birth defects globally [2, 3]. In China, the incidence of CHD is estimated to be between 8 and 10 per thousand live births based on birth defect surveillance data, indicating approximately 150,000 children born with CHD each year, with a mortality rate of 30% occurring within the first year of life [4]. Consequently, CHD represents a significant public health challenge affecting both maternal and child health.

The pathogenesis of CHD remains incompletely understood [5]. Clinical data indicate that genetic factors, including mutations and chromosomal aberrations, account for 9–18% of CHD cases, whereas approximately 80% are attributable to gene- environment interactions [6, 7]. Evidence suggests that several factors may increase the risk of CHD, such as advanced maternal age, lower socioeconomic status, occupational hazards, gestational diabetes mellitus, colds and fevers during early pregnancy, medication use, alcohol consumption, tobacco smoke, as well as exposure to environmental pollutants and air pollution [8–18]. Identifying potential causes of CHD, particularly environmental risk factors, is crucial for primary prevention.

Several predictive models for CHD have been developed. For instance, Huixia Li et al. constructed an artificial neural network model based on 15 predictors for CHD risk in a case-control study [19]. However, only 119 cases and 239 controls were included in their model. Besides, Yun Liang used the Hosmer-Lemeshow test and receiver operating characteristic (ROC) curve analysis to examine maternal risk factors for offspring CHD during pregnancy [20]. The sample sizes in these studies were relatively small, and no predictive tool for CHD was developed, limiting the utility of these models for clinical use. In this study, based on a comprehensive CHD database from Northwest China, we developed and validated a nomogram to predict the risk of CHD in the offspring of pregnant women.

## Methods

### Study design and participants

A survey on CHD was conducted among the population of Shaanxi province in 2013. The survey included pregnant women from the years 2010 to 2013 and covered nine cities within Shaanxi province. A standardized questionnaire was developed by the Xi’an Jiaotong University Health Science Center for this purpose. Trained field staff from the same institution conducted face-to-face interviews. The expected sample size was approximately 32,400 participants, and ultimately, 30,027 women completed the survey, resulting in a response rate of 92.68%.

From the survey, we collected data on maternal sociodemographic characteristics and periconceptional risk exposures. Additionally, information regarding the occurrence of CHD between enrollment and delivery, along with data on birth defects diagnosed at local hospitals, was also obtained. We excluded 823 individuals due to missing covariate information or unknown pregnancy outcomes, resulting in a total enrollment of 29,204 individuals for this study.

There were multi-discipline experts participating in the diagnosis of CHD in this study, including senior medical technicians from the departments of obstetrics and gynecology, ultrasound, and pediatric cardiac surgery at the first affiliated hospital of Xi’an Jiaotong University. To ensure the consistency and the accuracy of the diagnosis, all cases of CHD were diagnosed by these experts based on the International Statistical Classification of Diseases and Related Health Problems (ICD-10) coding system. For children identified with cardiovascular anomalies, their medical records were collected, and they underwent comprehensive ultrasound examinations free of charge at the first affiliated hospital of Xi’an Jiaotong University for the final diagnosis.

### Definitions of main variables

In this study, the primary outcome was the presence of CHD in the offspring. CHD was defined as a structural or functional abnormality of the heart that developed during fetal development and is present at birth, including conditions such as atrial septal defect, ventricular septal defect, patent ductus arteriosus, atrioventricular septal defect, tetralogy of Fallot, transposition of the great arteries, and hypoplastic left heart syndrome. For preterm infants who initially presented with atrial septal defects, persistent foramen ovale, and patent ductus arteriosus, follow-up assessments were conducted until 18 months of age to determine if these anomalies persisted, thereby allowing for the diagnosis of CHD.

A total of 14 factors were selected as predictive risk factors based on their variability and significance, including the gravidity (1, ≥ 2); pregnancy age (< 35 years, ≥ 35 years); abortion history (no, yes); preterm birth history (no, yes); family history of birth defects (no, yes); infection (no, yes), fever (no, yes), taking medicine (no, yes), alcohol consumption (no, yes), tobacco exposure (no, yes) in the early pregnancy; pesticide exposure in periconceptional period (no, yes); industries exposure in periconceptional period (no, yes); folic acid supplementation (no, yes); and singleton/twin pregnancy (singleton, twin).

The periconceptional period encompassed the three months before conception and the early stages of pregnancy (up to 12 weeks). Family history of birth defects was defined using congenital disabilities in immediate relatives. “Infection” referred to individuals who experienced at least one episode of flu or cold, or had a mild infection during early pregnancy. “Fever” was characterized by individuals who had a temperature exceeding 38 °C at least once during early pregnancy. “Taking medicine” included the use of any drugs such as antibiotics, anticancer agents, antidepressants, hormones, and other pharmaceuticals during early pregnancy. “Alcohol consumption” denoted women who consumed alcohol at least once during the early pregnancy period. “Tobacco exposure” was defined as women who smoked one cigarette per week for three consecutive months or were passively exposed to smoke for 15 min daily for one month during the periconceptional period. “Pesticide exposure” referred to pregnant women exposed to herbicides, fungicides, rodenticides, or insecticides during the periconceptional period. “Industrial exposure” included pregnant women residing within one kilometer of mines, fertilizer factories, cement factories, paper mills, pesticide factories, or power plants during pregnancy. “Folic acid supplementation” was defined as the daily consumption of 400 µg of folic acid for at least three consecutive months during the periconceptional period.

### Statistical analysis

70% of the subjects were randomly assigned to the training set for generating the nomogram, while the remaining 30% were allocated to the validation set for external verification. Both the training and validation sets were applied for the construction of the nomogram and external verification. Categorical variables were described using frequency and percentage, and differences among groups were assessed using the χ^2^ test. To identify factors associated with CHD, univariate logistic regression models were applied to the training set. Significant variables from the univariate analysis were included in a multivariate logistic regression model, with final model selected using a stepwise forward method. Prior to multivariate analysis, collinearity was assessed using contingency coefficients. Based on results from the multivariate logistic regression, a nomogram was developed to predict the risk of CHD using the selected variables. To evaluate the model’s discriminative performance, the AUC was determined using the C index. The calibration performance was evaluated using the chi-square of Hosmer-Lemeshow test. Additionally, the importance of predictive variables in random forest model was ranked using the mean decrease Gini method. Data analysis was conducted using *R software*, version 3.5.1. A two-tailed *p* < 0.05 was considered statistically significant.

## Results

### Patient demographics

A total of 29,204 women participated in the study, with 20,412 women assigned to the training group and the remaining 8,792 women assigned to the validation group. Table 1 presents the baseline characteristics of all enrolled subjects. The χ^2^ test revealed no significant differences in the characteristics of the participants between the training and validation groups.

**Table 1:** Basis characteristics of training group and validation group

Characteristics	Total (*N* = 29,204)	Training group (*N* = 20,412)	Validation group (*N* = 8792)	χ^2^	*P* value
Pregnancy age, n (%)				2.339	0.126
<35 years	26,935 (92.23)	18,794 (92.07)	8141 (92.60)		
≥ 35 years	2269 (7.77)	1618 (7.93)	651 (7.40)		
Gravidity, n (%)				0.037	0.847
1	14,926 (51.11)	10,440 (51.15)	4486 (51.02)		
≥ 2	14,278 (48.89)	9972 (48.85)	4306 (48.98)		
Abortion history, n (%)				1.020	0.313
no	25,147(86.11)	17,549 (85.97)	7598 (86.42)		
yes	4057 (13.89)	2863 (14.03)	1194 (13.58)		
Preterm birth history				1.663	0.197
no	28,388 (97.21)	19,825 (97.12)	8563 (97.40)		
yes	816 (2.79)	587 (2.88)	229 (2.60)		
Family history of birth defects, n (%)				2.215	0.137
no	29,068 (99.53)	20,309 (99.46)	8759 (99.62)		
yes	136 (0.47)	103 (0.50)	33 (0.38)		
Infection, n (%)				0.294	0.588
no	25,383 (86.92)	17,727 (86.85)	7656 (87.08)		
yes	3821 (13.08)	2685 (13.15)	1136 (12.92)		
Fever, n (%)				1.742	0.187
no	28,767 (98.50)	20,094 (98.44)	8673 (98.65)		
yes	437 (1.50)	318 (1.56)	119 (1.35)		
Taking medicine, n (%)				2.248	0.134
no	24,362 (83.42)	16,984 (83.21)	7378 (83.92)		
yes	4842 (16.58)	3428 (16.79)	1414 (16.08)		
Alcohol consumption, n (%)				1.393	0.238
no	28,869 (98.85)	20,168 (98.80)	8701 (98.96)		
yes	335 (1.15)	244 (1.20)	91 (1.04)		
Tobacco exposure, n (%)				0.389	0.533
no	11,845 (40.56)	8255 (40.44)	3590 (40.83)		
yes	17,359 (59.44)	12,157 (59.56)	5202 (59.17)		
Pesticide exposure, n (%)				0.234	0.629
no	28,865 (98.84)	20,171 (98.82)	8694 (98.89)		
yes	339 (1.16)	241 (1.18)	98 (1.11)		
Industries exposure, n (%)				0.214	0.644
no	21,438 (73.41)	15,000 (73.49)	6438 (73.23)		
yes	7766 (26.59)	5412 (26.51)	2354 (26.77)		
Optimal folic acid supplementation, n (%)				0.012	0.914
no	18,611 (63.73)	13,004 (63.71)	5607 (63.77)		
yes	10,593 (36.27)	7408 (36.29)	3185 (36.23)		
Singleton/twin pregnancy, n (%)				0.487	0.485
singleton	28,852 (98.79)	20,160 (98.77)	8692 (98.86)		
twin	352 (1.21)	252 (1.23)	100 (1.14)		
CHD, n (%)				2.957	0.085
no	29,019 (99.37)	20,272 (99.31)	8747 (99.49)		
yes	185 (0.63)	140 (0.69)	45 (0.51)		

### Nomogram development

Table 2 summarizes the results of univariate and multivariate analyses conducted on the training set for CHD and potential predictive risk factors. Results showed that there were eight significant predictive risk factors for CHD, including the gravidity, preterm birth history, family history of birth defects, infection, taking medicine, tobacco exposure, pesticide exposure and singleton/twin pregnancy.

Table 2 presents the results of the multivariate logistic regression model. We observed that the risk of CHD were significantly associated with the following predictors: gravidity ≥ 2 (OR:1.46, 95% CI:1.04, 2.07), preterm birth history (OR: 2.32, 95% CI: 1.21, 4.46), family history of birth defects (OR: 3.91, 95% CI: 1.21, 12.68), infection (OR: 2.42, 95% CI: 1.67, 3.52), taking medicine (OR: 2.34, 95% CI: 1.64, 3.35), tobacco exposure (OR: 1.63, 95% CI: 1.12, 2.37), pesticide exposure (OR: 3.94, 95% CI: 1.95, 7.96), and twin pregnancy (OR: 3.09, 95% CI: 1.30, 7.37).

**Fig. 1:**
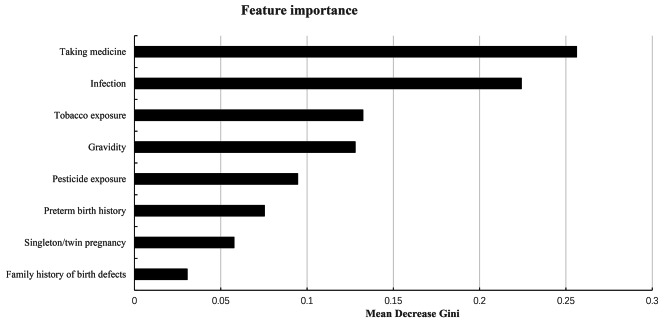
The importance ranking of factors related to CHD, including gravidity, preterm birth history, family history of birth defects, infection, taking medicine, tobacco exposure, pesticide exposure and singleton/twin pregnancy. The larger the mean decrease Gini, the more important the indicator was

Based on the random forest algorithm, the importance of predictor variables was ranked (Fig. 1). A higher mean decrease in Gini coefficient indicated greater variable importance. Taking medicine emerged as the most significant predictive risk factor, followed by infection, tobacco exposure, gravidity, pesticide exposure, preterm birth history, singleton/twin pregnancy, and family history of birth defects.

**Fig. 2:**
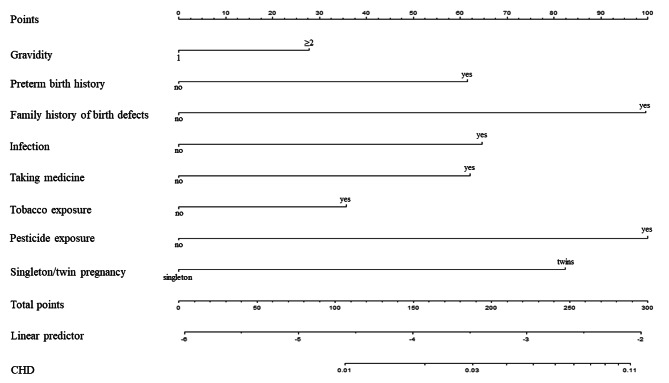
Nomogram for predicting CHD

**Fig. 3:**
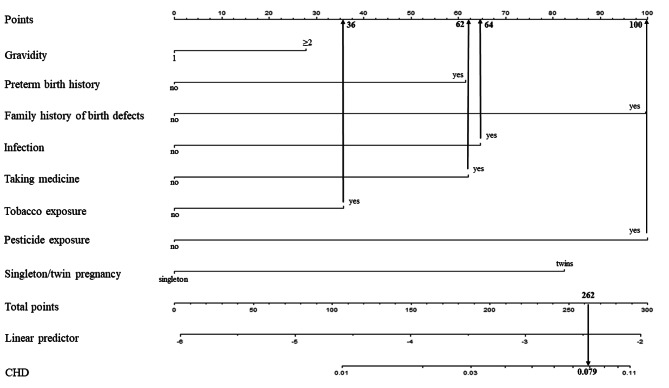
Example prediction nomogram for risk of CHD.

According to the multivariate logistic regression model with eight independent predictive risk factors, we developed an individualized nomogram model for the prediction of CHD (Fig. 2). The nomogram assigned a specific score to each predictive risk factor, and the total score was calculated by summing these individual scores. The predicted risk of CHD was then determined based on the corresponding probability associated with the total score. For example, a pregnant woman who scored 0 points when she was in her first pregnancy with no preterm birth history, no family history of birth defects, and a singleton pregnancy. If this woman also experienced infection, taking medicine, tobacco exposure, and pesticide exposure, she would accumulate 64, 62, 36, and 100 points, respectively. Therefore, the total score would be 64 + 62 + 36 + 100 = 262. According to Fig. 3, the predicted risk of CHD in her offspring would be 0.079 (79‰).

**Table 2:** Univariate and multivariate logistic analysis of factors predicting CHD in the training group

Variables	Univariate logistic analysis			Multivariate logistic analysis	
	OR (95%CI)	*P* value		OR (95%CI)	*P* value
Pregnancy age, n (%)					
<35 years	1.00				
≥ 35 years	1.40 (0.82, 2.39)	0.223			
Gravidity, n (%)					
1	1.00			1.00	
≥ 2	1.67 (1.19, 2.35)	0.003		1.46 (1.04, 2.07)	0.021
Abortion history, n (%)					
no	1.00				
yes	1.83 (1.23, 2.71)	0.003			
Preterm birth history, n (%)					
no	1.00			1.00	
yes	2.92 (1.57, 5.43)	0.001		2.32 (1.21, 4.46)	0.011
Family history of birth defects, n (%)					
no	1.00			1.00	
yes	4.42 (1.38, 14.10)	0.012		3.91 (1.21, 12.68)	0.023
Infection, n (%)					
no	1.00			1.00	
yes	3.16 (2.21, 4.52)	< 0.001		2.42 (1.67, 3.52)	< 0.001
Fever, n (%)					
no	1.00				
yes	2.87 (1.26, 6.54)	0.012			
Taking medicine, n (%)				1.00	
no	1.00			2.34 (1.64, 3.35)	< 0.001
yes	3.15 (2.23,4.43)	< 0.001			
Alcohol consumption, n (%)					
no	1.00				
yes	1.20 (0.30, 4.87)	0.799			
Tobacco exposure, n (%)					
no	1.00			1.00	
yes	1.77 (1.22, 2.56)	0.003		1.63 (1.12, 2.37)	0.010
Pesticide exposure, n (%)					
no	1.00			1.00	
yes	5.93 (2.98, 11.80)	< 0.001		3.94 (1.95, 7.96)	< 0.001
Industries exposure, n (%)					
no	1.00				
yes	1.27 (0.89, 1.82)	0.187			
Optimal folic acid supplementation, n (%)					
no	1.00				
yes	0.73 (0.50, 1.05)	0.085			
Singleton/twin pregnancy, n (%)					
singleton	1.00			1.00	
twin	3.65 (1.59,8.34)	0.002		3.09 (1.30, 7.37)	0.011

### Validation of Nomogram

**Fig. 4:**
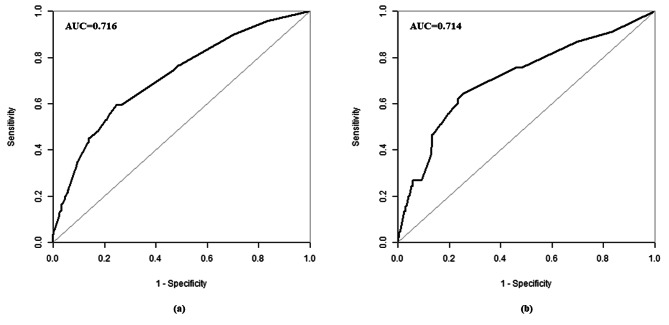
ROC curve in training group (a) and validation group (b)

**Fig. 5:**
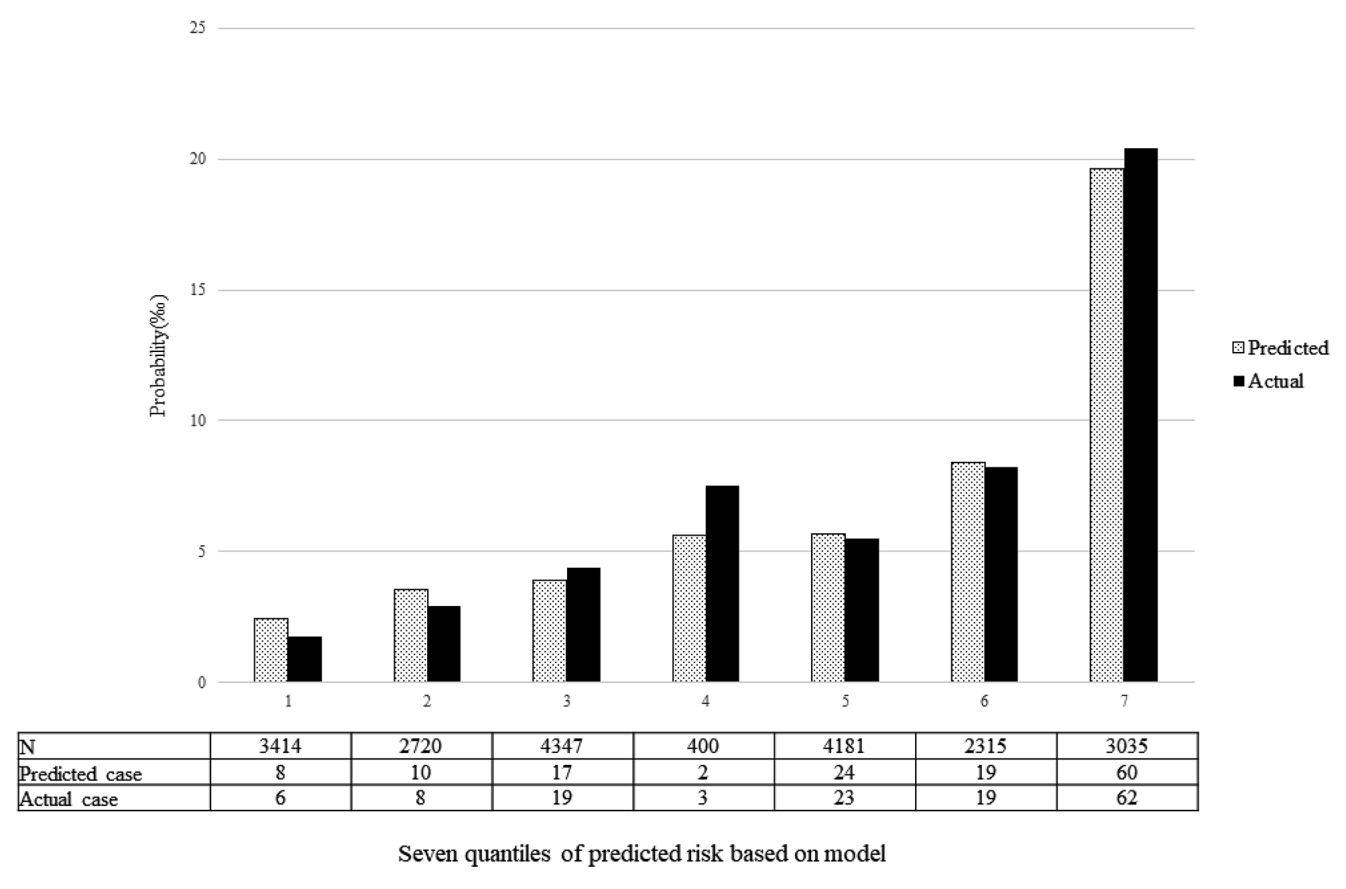
Calibration plot. The x-axis represents quintiles of predicted risk, and the y-axis reveals predicted and actual probability of CHD

The validation of the predictive model used both calibration and discrimination methods. For this process, ROC curves for the predicted probability were constructed for both the validation and training groups, and the AUC values were calculated. Using the ROC curve, the AUC values for the nomogram, which included 8 independent predictive risk factors, were determined to be 0.714 (95% CI: 0.630, 0.798) for the validation group and 0.716 (95% CI: 0.671, 0.760) for the training group, as shown in Table 3; Fig. 4. These AUC values indicated that the nomogram prediction model might possess moderate discrimination ability. The optimal threshold for the model was identified as 0.0076, at which the model achieved a sensitivity of 59.29%, a specificity of 75.39%, and an accuracy of 75.28%. Furthermore, the chi-square of Hosmer-Lemeshow test was calculated to be 1.529 with a *p*-value of 0.910, indicating good calibration of the predictive model (Fig. 5).

**Table 3:** The AUCs of the ROC curves for the nomogram and variables from the logistic regression model in the training group and validation group

	Training group				Validation group		
	AUC	95% CI	*P* value		AUC	95% CI	*P* value
Nomogram variable	0.716	0.671–0.760	< 0.001		0.714	0.630–0.798	< 0.001
Gravidity	0.563	0.516–0.610	0.010				
Preterm birth history	0.525	0.475–0.575	0.306			-	
Family history of birth defects	0.508	0.460–0.557	0.736			-	
Infection	0.596	0.544–0.648	< 0.001			-	
Taking medicine	0.610	0.558–0.661	< 0.001			-	
Tobacco exposure	0.563	0.518–0.609	0.010				
Pesticide exposure	0.526	0.476–0.577	0.281			-	
Singleton/twin pregnancy	0.515	0.466–0.565	0.530			-	

## Discussion

This study developed an innovative predictive model for assessing the risk of CHD, contrasting with the predominant research emphasis on its etiology. A total of 29,204 pregnant women from Shaanxi province of China were included in this study, representing the largest cohort for the development of the nomogram to predict the risk of CHD. Finally, we identified eight predictive risk factors of CHD, including the gravidity, preterm birth history, family history of birth defects, infection, taking medicine, tobacco exposure, pesticide exposure and singleton/twin pregnancy.

CHD is the most prevalent congenital malformation, accounting for approximately one-third of all congenital anomalies [21]. Between 1970 and 2017, the global birth prevalence of CHD has steadily increased by 10% every five years, peaking at 9.41 per 1000 births [3]. The prevalence of CHD in Asia was higher compared to Europe and America [3]. Given China’s large population, CHD has emerged as a significant maternal and child health concern. Screening for risk factors and assessing risk levels are crucial for the population-based primary prevention of CHD [22, 23].

Several predictive models for CHD have been developed in previous research. Yun Liang et al. constructed a logistic regression model using four key predictive factors—respiratory infections, polluted water exposure, adverse emotions during pregnancy, and nutrient deficiencies—to predict the risk of CHD in the offspring of pregnant women. The model achieved an AUC of 0.72 (95% CI: 0.681, 0.759) [20]. In another study, Huixia Li et al. identified 15 predictive risk factors of CHD using univariate logistic regression analyses and developed a prediction model based on a feed-forward back-propagation neural network (BPNN). Their model demonstrated an AUC of 0.87 [19]. However, these models were derived from small sample case-control studies, which may limit their generalizability to real-world populations. In contrast, our CHD prediction model was based on a large-scale CHD survey, resulting in a robust nomogram designed as a predictive application tool for CHD.

Our study identified significant associations between twin pregnancy, family history of birth defects, preterm birth history, and gravidity with an increased risk of CHD. Epidemiological evidence has consistently reported associations between pregnancy history and twin pregnancies with CHD risk. Yu et al. conducted a systematic review and meta-analysis, revealing a summary OR of 1.13 (95% CI: 1.08, 1.18) for each additional pregnancy in relation to the risk of CHD [24]. They also found that a family history of birth defects increased the risk of CHD by 314% (OR:4.14, 95% CI: 2.47, 6.96) [25]. Moreover, population-based data from the Northern Congenital Abnormality Survey in England between 1998 and 2010 indicated that twins have a 73% higher likelihood of CHD compared to singletons (RR: 1.73, 95% CI: 1.48, 2.04) [26]. Our findings were consistent with these previous investigations, underscoring the significance of these predictive factors in the risk assessment of CHD.

Our study revealed that the risk of CHD was linked to various diseases, adverse health behaviors, and environmental factors, including infection, medication use, tobacco consumption, and pesticide exposure. These findings were consistent with existing literature. For instance, a case-control study demonstrated that the increased risk of CHD were associated with upper respiratory tract infections (OR: 3.40, 95% CI: 2.05, 5.62) and influenza (OR: 2.39, 95% CI: 1.47, 3.88) during early pregnancy [27]. Other studies have reported increased risk of CHD in the offspring related to the use of antidepressants and antiepileptics during pregnancy [28, 29]. According to a population-based matched case-control study involving 9,452 subjects, maternal smoking during the first trimester was significantly associated with an increased likelihood of CHD in infants (OR: 1.44, 95% CI: 1.25, 1.66) in a dose-response manner [30]. Moreover, a multisite case-control study indicated that exposure to fungicides, insecticides, and herbicides might elevate the risk of specific CHD subtypes, such as secundum atrial septal defect, hypoplastic left heart syndrome, and tetralogy of Fallot [31].

The predictive model serves as a valuable tool for obstetricians in assessing the risk of CHD in the fetus during early pregnancy. In cases where elevated risk is identified, it is advisable for pregnant women to consult with a fetal cardiologist and undergo fetal heart ultrasound examination during mid-pregnancy. Furthermore, pregnant women should receive information and education regarding birth defects, along with guidance on reducing the risk of CHD through lifestyle adjustments, including the supplementation of folic acid.

Despite the progress achieved in this project, several limitations should be acknowledged. Firstly, all the data of this study came from a survey, potentially introducing recall bias into our results. To mitigate this bias, we implemented a stringent investigation protocol and selected clear indices related to exposure, thereby aiding participants in the accurate recalling of long-term exposure histories. For instance, the indices including detailed questionnaires covering common diseases, types of medications, and their specific names during the periconceptional period were adopted in this study. Additionally, we cross-checked responses against any available paper medical records to minimize the impact of recall bias. Secondly, our study may have overlooked some predictive risk factors that could influence the value of C index. Factors such as maternal obesity (pre-pregnancy and early pregnancy), pregestational diabetes mellitus, gestational diabetes, pre-existing hypertension, and genes related to folate metabolism have been reported to be associated with the risk of CHD in previous studies. These variables were not included in our current model, suggesting that future research could incorporate additional prognostic factors to improve the predictive capability of our model. Finally, our prediction model was developed based on data from the Northwest Chinese population. Therefore, caution should be exercised when extrapolating these findings to other populations or geographic regions, as demographic and environmental factors can vary significantly.

## Conclusion

In conclusion, we have developed an individualized nomogram to predict the risk of CHD in the offspring of pregnant women based on identified prenatal predictive risk factors. It would be a potential tool for the early gestational assessment of the risk of CHD during early pregnancy and for the delivery of targeted health education. To validate the efficacy and applicability of our nomogram, additional prospective birth cohort studies are warranted in future.

## Data Availability

The raw data supporting the conclusions of this article will be made available by the authors, without undue reservation.
